# Real-world effectiveness of first-line immunotherapy with or without chemotherapy versus chemotherapy alone in advanced non-small cell lung cancer

**DOI:** 10.3389/fimmu.2026.1753591

**Published:** 2026-05-12

**Authors:** Lihua Li, Juan Du, Jing Yang, Limin Chai, Jingxu Nie, Xinfeng Cai, Linxiu He, Junting Jia, Rong Ma, Yimeng Guo

**Affiliations:** Department of Pharmacy, Shanxi Province Cancer Hospital/Shanxi Hospital Affiliated to Cancer Hospital, Chinese Academy of Medical Sciences/Cancer Hospital Affiliated to Shanxi Medical University, Taiyuan, Shanxi, China

**Keywords:** immune checkpoint inhibitors, immunotherapy, non-small cell lung cancer, programmed death ligand-1, programmed death receptor-1

## Abstract

This real-world retrospective study evaluated the effectiveness of first-line immunotherapy with or without chemotherapy compared with chemotherapy alone in patients with advanced non-small cell lung cancer (NSCLC). It also explored survival outcomes associated with domestically developed and internationally developed PD-1 inhibitors in China.

**Methods:**

Data were collected from 401 patients with stage IV NSCLC treated at Shanxi Province Cancer Hospital between January 2019 and December 2021, including 248 in the chemotherapy group and 153 in the immune checkpoint inhibitors (ICIs) with or without chemotherapy group. The primary outcome was overall survival (OS). Kaplan-Meier curves and log-rank tests were used to evaluate survival differences, and Cox proportional hazards models were employed for subgroup analyses.

**Results:**

The median OS was significantly longer in the ICIs with or without chemotherapy group than in the chemotherapy group (22.0 months vs. 14.0 months, *p* < 0.0001). In a propensity score-matched cohort of 236 patients, ICIs with or without chemotherapy remained associated with significantly longer OS than chemotherapy alone. Exploratory subgroup analyses suggested potential heterogeneity in the survival benefit associated with ICIs-based treatment according to BMI, pre-existing pulmonary disease, smoking status, and T stage; however, these findings should be interpreted cautiously given the limited sample sizes within some subgroups. Concomitant corticosteroid use was associated with shorter OS (median 20.0 vs. 26.0 months, *p* = 0.036), while antibiotic and proton pump inhibitor use showed non-significant trends toward reduced survival. No significant OS difference was observed between domestically developed ICIs (camrelizumab, sintilimab, tislelizumab) and the internationally developed agent pembrolizumab (median 22.0 vs. 24.0 months, *p* = 0.28).

**Conclusion:**

First-line immunotherapy with or without chemotherapy significantly improves survival compared with chemotherapy alone in advanced NSCLC. No statistically significant difference in overall survival was observed between domestically developed PD-1 inhibitors and pembrolizumab in this cohort; however, this comparison should be interpreted cautiously given the limited sample size, particularly in the internationally developed ICIs group.

## 1 Introduction

Lung cancer is one of the most prevalent and deadly malignancies worldwide. In 2022 alone, it caused approximately 1.8 million deaths, posing a severe threat to global health (1). According to epidemiological statistics, China witnessed approximately 870,000 new lung cancer cases and 770,000 related deaths in 2022, ranking it first in both incidence and mortality rates among all malignancies. Notably, non-small cell lung cancer (NSCLC) accounted for about 80% of these cases (2). Due to the absence of apparent early symptoms, many patients are diagnosed at an advanced stage, which contributes to the persistently high mortality rate.

The treatment landscape for driver-negative advanced NSCLC has undergone a profound transformation over the past two decades owing to the introduction of immunotherapy. This transformation is exemplified by the approval by China’s National Medical Products Administration (NMPA) of several programmed death receptor-1 (PD-1) and programmed death ligand-1 (PD-L1) inhibitors for both first- and second-line treatment, either as monotherapy or in combination with chemotherapy. These agents include nivolumab, pembrolizumab, camrelizumab, sintilimab, tislelizumab, and toripalimab. PD-1 is an immune checkpoint receptor expressed on T cells, while PD-L1 is its corresponding ligand located on tumor cells. The PD-1/PD-L1 interaction suppresses T-cell activation and proliferation, thereby enabling tumor cells to evade immune surveillance (3–5).

Major clinical trials—including KEYNOTE-024 (6, 7), KEYNOTE-042 (8, 9), IMpower130 (10), KEYNOTE-189 (11), and KEYNOTE-407 (12)—have demonstrated that immune checkpoint inhibitors (ICIs), when used either as monotherapy or in combination with chemotherapy, significantly prolong overall survival compared with chemotherapy alone in patients with advanced NSCLC. However, these findings are primarily derived from randomized clinical trials (RCTs) that employ highly selective inclusion and exclusion criteria. In contrast, real-world clinical practice involves a more heterogeneous and complex patient population. Consequently, meta-analyses based solely on RCT data may not fully capture treatment outcomes observed in routine clinical settings. Therefore, real-world evidence is essential to better characterize the effectiveness of ICIs-based therapies in broader patient populations.

Our team conducted a real−world, retrospective study to investigate the effectiveness of first−line ICIs therapy in patients with stage IV lung cancer. Against this background, and given the increasing clinical adoption of domestically developed PD−1 inhibitors in China—such as camrelizumab, sintilimab, and tislelizumab—it has become important to determine whether their therapeutic efficacy is comparable to that of pembrolizumab, the internationally recognized benchmark. Nevertheless, direct head−to−head evidence comparing these agents in real−world settings remains scarce. To address this knowledge gap, the present study further evaluated the comparative efficacy of three Chinese−developed PD−1 inhibitors (camrelizumab, sintilimab, and tislelizumab) versus pembrolizumab in patients with advanced NSCLC. The findings aim to provide clinically relevant evidence on the real−world effectiveness of these immunotherapies within the Chinese lung cancer population.

## 2 Materials and methods

### 2.1 Patient population and data collection

This retrospective study was conducted at Shanxi Province Cancer Hospital using data extracted from the institution’s electronic medical record (EMR) system. Survival information was obtained from the hospital’s affiliated follow−up center. Patient data were collected between January 2019 and December 2021.

Patients meeting the following criteria were included in the study: (1) aged over 18 years; (2) pathologically confirmed diagnosis of stage IV NSCLC; (3) presence of measurable disease according to the Response Evaluation Criteria in Solid Tumors (RECIST) version 1.1; (4) receipt of either chemotherapy alone or immunotherapy with or without chemotherapy (immunotherapy ± chemotherapy) as first−line treatment; (5) completion of at least four cycles of systemic therapy; and (6) availability of documented follow−up or death dates. Exclusion criteria were as follows: (1) prior history of antineoplastic treatment; (2) presence of targetable gene mutations such as *EGFR, ALK*, or *ROS1*; (3) prior or concurrent diagnosis of other malignancies; and (4) incomplete clinical data or loss to follow−up. This study received ethical approval from the Shanxi Province Cancer Hospital Ethical Review Board (No. KY2024053). Due to the retrospective design of the study, the requirement for informed consent was waived by the ethics committee.

The primary outcome of the study was overall survival (OS), defined as the time from the date of pathological diagnosis of the tumor to death from any cause or the end of follow-up, whichever occurred first. The follow-up period ended on 30 November 2024. By reviewing the patients’ medical records, we collected demographic and clinical characteristics, including age, sex, comorbidities, body mass index (BMI), NRS-2002 nutritional risk score, histologic type of tumor, TNM stage, sites of metastases (brain, liver, and bone), PD-L1 expression status, treatment regimen, and smoking and alcohol consumption habits. To evaluate the potential influence of concomitant medications on clinical outcomes, three categories of drugs were analyzed: antibiotics, proton pump inhibitors, and systemic corticosteroids. Use of these medications was uniformly defined as exposure to the respective drug within 30 days before or after initiation of ICIs therapy. For systemic corticosteroids, exposure was defined as oral or intravenous administration at a dose equivalent to ≥10 mg prednisone per day within this timeframe, regardless of duration.

### 2.2 Statistical analysis

Continuous variables were summarized as mean (SD) when normally distributed or as median (IQR) otherwise, and between-group differences were assessed using independent-samples *t-*tests or Wilcoxon rank-sum tests. Categorical variables were presented as number (percentage) and compared using *χ*² tests. OS was estimated with Kaplan-Meier curves and compared between groups using the log-rank test. A two-sided *α* level of 0.05 was predefined, and *p*-values below this threshold were deemed statistically significant.

To further reduce potential treatment selection bias and assess the robustness of the primary findings, a propensity score matching analysis was performed as a sensitivity analysis. Propensity scores were estimated using a logistic regression model based on baseline covariates measured before treatment initiation, including age, sex, BMI, nutritional status, smoking status, drinking status, diabetes, cardiovascular disease, lung disease, T stage, N stage, histological type, liver metastasis, bone metastasis, brain metastasis, and PD-L1 expression1. Patients in the chemotherapy group and the ICIs ± chemotherapy group were matched 1:1 using nearest-neighbor matching with a caliper width of 0.25. Overall survival was then re-evaluated in the matched cohort using Kaplan-Meier curves and the log-rank test.

To identify factors independently associated with OS, univariable Cox proportional hazards regression analyses were first performed for relevant clinical variables. Variables with p < 0.1 in the univariable analyses were subsequently included in the multivariable Cox proportional hazards regression model. The multivariable model was constructed using the enter method, with all selected variables entered simultaneously. Hazard ratios (HRs) and 95% confidence intervals (CIs) were reported.

To explore potential heterogeneity in treatment effects across patient subgroups, subgroup analyses were further conducted using multivariable Cox proportional hazards regression models. Variables with potential prognostic or confounding effects, including baseline-imbalanced variables, were included as covariates for adjustment. Subgroup analyses were performed according to prespecified clinical and demographic characteristics. To assess whether the association between treatment and OS differed across subgroups, treatment-by-subgroup interaction terms were incorporated into the Cox models, and effect modification was evaluated using tests for interaction. All statistical analyses were performed using R software (version 4.2.1).

## 3 Results

### 3.1 Characteristics of study patients

A total of 401 patients with advanced NSCLC were included, comprising 248 in the chemotherapy group and 153 in the ICIs ± chemotherapy group. The majority were male and aged ≥ 60 years. Most patients had a normal BMI and good nutritional status. Compared with the chemotherapy group, the ICIs ± chemotherapy group included a higher proportion of patients with advanced N stage (*p* = 0.017) and PD−L1 ≥ 50% expression (*p* < 0.001). This pattern likely reflects clinical practice preferences, as patients with positive or high PD−L1 expression are generally more likely to receive immunotherapy. The ICIs ± chemotherapy group also had a longer median follow−up duration (22.0 months vs 14.0 months, *p* < 0.001), and a greater proportion of patients remained alive at the end of follow−up (22.9% vs 5.2%, *p* < 0.001). Other baseline characteristics were generally well balanced between the two groups. Among the 153 patients in the ICIs ± chemotherapy group, 10 received immunotherapy alone, and 143 received immunotherapy combined with chemotherapy. Therefore, the overall findings for the pooled ICIs-based group were driven predominantly by patients receiving chemo-immunotherapy. Specifically, among the 143 patients who received immunotherapy combined with chemotherapy, 82 received pemetrexed plus platinum, 54 received taxanes plus platinum, and 7 received gemcitabine plus platinum. Details are shown in **Table 1**.

**Table 1:** Clinical characteristics of patients with advanced NSCLC.

Characteristics	[ALL] N=401	Chemotherapy N=248	ICIs ± chemotherapy N=153	*P* value
Sex, n (%)				0.166
Female	81 (20.2%)	56 (22.6%)	25 (16.3%)	
Male	320 (79.8%)	192 (77.4%)	128 (83.7%)	
Age(years), n (%)				0.100
<60	150 (37.4%)	101 (40.7%)	49 (32.0%)	
≥60	251 (62.6%)	147 (59.3%)	104 (68.0%)	
BMI, n (%)				0.214
<18.5	24 (6.0%)	11 (4.4%)	13 (8.5%)	
18.5-24.9	265 (66.1%)	169 (68.1%)	96 (62.7%)	
>25	112 (27.9%)	68 (27.4%)	44 (28.8%)	
Nutrition Score, n (%)				1.000
<3	346 (86.3%)	214 (86.3%)	132 (86.3%)	
≥3	55 (13.7%)	34 (13.7%)	21 (13.7%)	
Smoking, n (%)				0.120
No	119 (29.7%)	81 (32.7%)	38 (24.8%)	
Yes	282 (70.3%)	167 (67.3%)	115 (75.2%)	
Drinking, n (%)				0.940
No	231 (57.6%)	142 (57.3%)	89 (58.2%)	
Yes	170 (42.4%)	106 (42.7%)	64 (41.8%)	
Diabetes comorbidity, n (%)				0.320
No	365 (91.0%)	229 (92.3%)	136 (88.9%)	
Yes	36 (9.0%)	19 (7.7%)	17 (11.1%)	
Cardiovascular diseases comorbidity, n (%)				0.951
No	306 (76.3%)	190 (76.6%)	116 (75.8%)	
Yes	95 (23.7%)	58 (23.4%)	37 (24.2%)	
Lung Disease Comorbidity, n (%)				0.330
No	313 (78.1%)	198 (79.8%)	115 (75.2%)	
Yes	88 (21.9%)	50 (20.2%)	38 (24.8%)	
T stage, n (%)				0.666
1-2	195 (48.6%)	118 (47.6%)	77 (50.3%)	
3-4	206 (51.4%)	130 (52.4%)	76 (49.7%)	
N stage, n (%)				0.040
0-1	77 (19.2%)	56 (22.6%)	21 (13.7%)	
2-3	324 (80.8%)	192 (77.4%)	132 (86.3%)	
Histological type, n (%)				0.625
Non-squamous	253 (63.1%)	157 (63.3%)	96 (62.7%)	
Squamous	136 (33.9%)	82 (33.1%)	54 (35.3%)	
Unknown	12 (3.0%)	9 (3.6%)	3 (1.96%)	
Liver metastasis, n (%)				1.000
No	347 (86.5%)	215 (86.7%)	132 (86.3%)	
Yes	54 (13.5%)	33 (13.3%)	21 (13.7%)	
Bone metastasis, n (%)				0.489
No	238 (59.4%)	151 (60.9%)	87 (56.9%)	
Yes	163 (40.6%)	97 (39.1%)	66 (43.1%)	
Brain metastasis, n (%)				0.529
No	312 (77.8%)	196 (79.0%)	116 (75.8%)	
Yes	89 (22.2%)	52 (21.0%)	37 (24.2%)	
PD-L1 expression, n (%)				<0.001
<1%	50 (12.5%)	30 (12.1%)	20 (13.1%)	
1% ≤PD-L1 < 50%	44 (11.0%)	17 (6.9%)	27 (17.6%)	
PD-L1≥50%	57 (14.2%)	14 (5.7%)	43 (28.1%)	
Unknown	250 (62.3%)	187 (75.4%)	63 (41.2%)	
Dead, n (%)				<0.001
No	48 (12.0%)	13 (5.2%)	35 (22.9%)	
Yes	353 (88.0%)	235 (94.8%)	118 (77.1%)	
Time, Median (IQR)	17.0 [9.00;30.0]	14.0 [8.00;25.0]	22.0 [12.0;37.0]	<0.001

### 3.2 Survival outcomes

**Figure 1:**
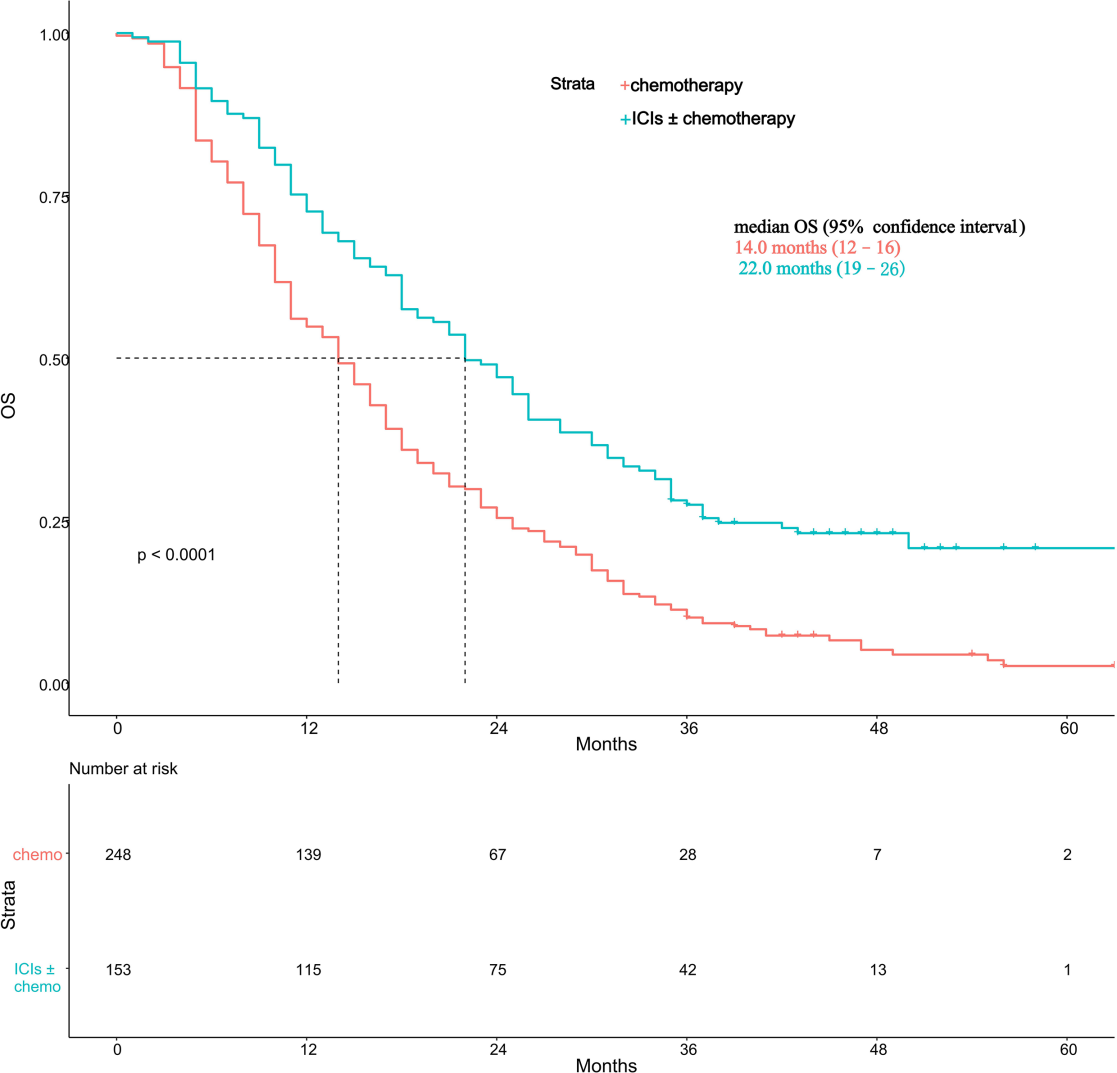
Kaplan-Meier overall survival curves for the chemotherapy group and the ICIs ± chemotherapy group. Chemo, chemotherapy.

The Kaplan-Meier analysis revealed a significant difference in OS between the two treatment groups (*p* < 0.0001, **Figure 1**). The median OS was 14.0 months (95% CI, 12-16) in the chemotherapy group and 22.0 months (95% CI, 19-26) in the ICIs± chemotherapy group. Patients receiving ICIs ± chemotherapy experienced significantly longer survival compared with those receiving chemotherapy alone.

Multivariable Cox proportional hazards regression analysis showed that T stage (HR = 1.320, 95% CI: 1.060-1.643, P = 0.013), bone metastasis (HR = 1.525, 95% CI: 1.224-1.900, P < 0.001), and first-line treatment regimen (HR = 0.578, 95% CI: 0.454-0.738, P < 0.001) were independent prognostic factors for OS. Specifically, a more advanced T stage and the presence of bone metastasis were associated with poorer overall survival, whereas receipt of ICIs-based first-line therapy was associated with improved overall survival. Details are shown in **Table 2**.

**Table 2:** Univariable and multivariable Cox proportional hazards regression analyses for overall survival.

Variable	Univariate analysis			Multivariate analysis		
	HR	CI	P-value	HR	CI	P-value
First line treatment (Chemotherapy vs. Immunotherapy)	0.54	0.432-0.675	0	0.578	0.454-0.738	< 0.001
PD-L1 expression						
PD-L1 <1% vs. 1% ≤PD-L1 < 50%	0.793	0.51-1.232	0.303	0.911	0.583-1.422	0.681
PD-L1 <1% vs. PD-L1≥50%	0.677	0.445-1.029	0.068	0.868	0.562-1.339	0.521
PD-L1 <1% vs. Unknown	1.089	0.792-1.495	0.6	1.078	0.784-1.484	0.644
Brain metastasis (No vs. Yes)	1.221	0.951-1.567	0.117			
Bone metastasis (No vs. Yes)	1.437	1.163-1.776	0.001	1.525	1.224-1.900	< 0.001
Liver metastasis (No vs. Yes)	1.306	0.97-1.758	0.078	1.22	0.899-1.656	0.202
Histological type						
Non-squamous vs. Squamous	1.078	0.864-1.346	0.507			
Non-squamous vs. Unknown	1.307	0.731-2.338	0.367			
N (<N2 vs. ≥N2)	1.249	0.954-1.635	0.107			
T (<T3 vs. ≥T3)	1.261	1.02-1.558	0.032	1.32	1.06-1.643	0.013
Lung disease comorbidity (No vs. Yes)	0.938	0.724-1.216	0.629			
Cardiovascular diseases comorbidity (No vs. Yes)	0.919	0.717-1.179	0.507			
Diabetes comorbidity (No vs. Yes)	0.85	0.588-1.229	0.388			
Drinking (No vs. Yes)	1.082	0.876-1.338	0.465			
Smoking (No vs. Yes)	1.003	0.799-1.259	0.98			
Nutrition score (<3 vs. ≥3)	1.056	0.784-1.421	0.721			
BMI						
BMI1<18.5 vs. 18.5≤BMI ≤ 24.9	1.14	0.722-1.8	0.573			
BMI1<18.5 vs. BMI ≥25	0.94	0.58-1.524	0.802			
Age (<60 vs. ≥60)	1.086	0.875-1.349	0.453			
Sex (Female vs. Male)	1.136	0.875-1.474	0.338			

To further validate the main findings, a propensity score-matched analysis was conducted. After 1:1 nearest-neighbor matching with a caliper of 0.25, 236 patients were retained in the matched cohort, including 118 patients in the chemotherapy group and 118 patients in the ICIs ± chemotherapy group. Details are shown in **Supplementary Table 1**. Kaplan-Meier analysis in the matched cohort continued to show significantly longer overall survival in the ICIs ± chemotherapy group than in the chemotherapy group (*p* < 0.0001). The median OS was 14.0 months (95% CI, 11-17) in the chemotherapy group and 21.5 months (95% CI, 18-26) in the ICIs ± chemotherapy group (**Supplementary Figure S1**). These findings were consistent with the results of the original cohort, supporting the robustness of the survival benefit associated with ICIs-based treatment.

### 3.3 Subgroup analysis

**Figure 2:**
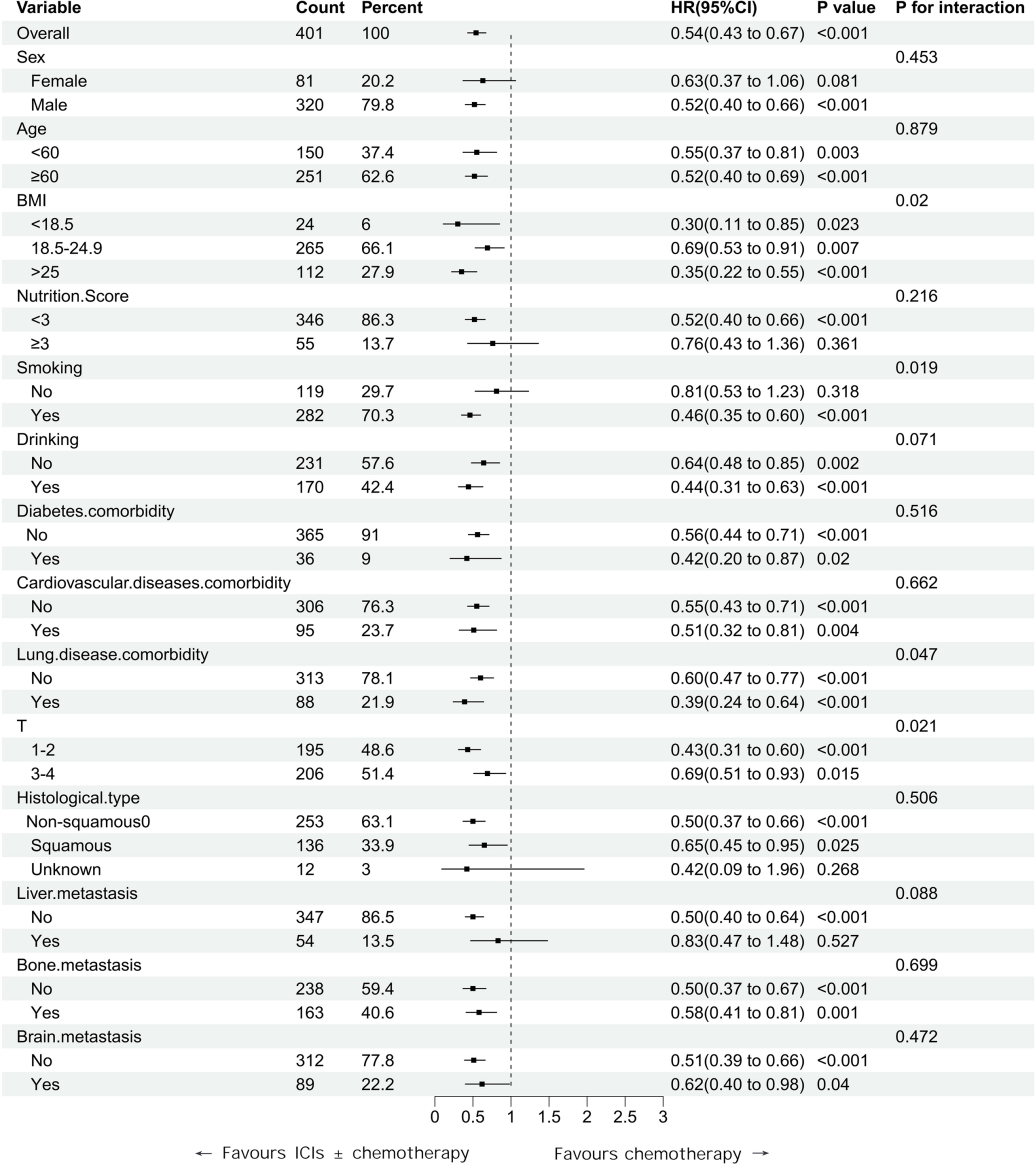
Subgroup analysis of overall survival comparing ICIs ± chemotherapy with chemotherapy.

Given the improved overall survival observed in patients receiving ICIs ± chemotherapy compared with chemotherapy alone, we further examined whether this treatment effect was consistent across clinical subgroups. Since N stage and PD-L1 expression differed significantly between treatment groups at baseline, both variables were included as covariates in the multivariable models rather than used as stratification factors. Subgroup analyses were therefore adjusted for these variables and conducted according to baseline demographics and clinical characteristics, including sex, age, comorbidities, BMI, nutritional status score, smoking history, and T stage (**Figure 2**).

The survival benefit associated with ICIs ± chemotherapy was generally consistent across most subgroups. Exploratory analyses identified statistically significant treatment-by-subgroup interactions for BMI, pre-existing pulmonary disease, smoking status, and T stage. The relative survival benefit of ICIs ± chemotherapy appeared greater in patients with abnormal BMI, pre-existing pulmonary disease, a history of smoking, and earlier T-stage tumors. However, given the retrospective design and the limited sample sizes within several subgroups, these findings should be considered hypothesis-generating rather than definitive evidence of treatment-effect heterogeneity.

### 3.4 Influence of concomitant medications on overall survival

**Figure 3:**
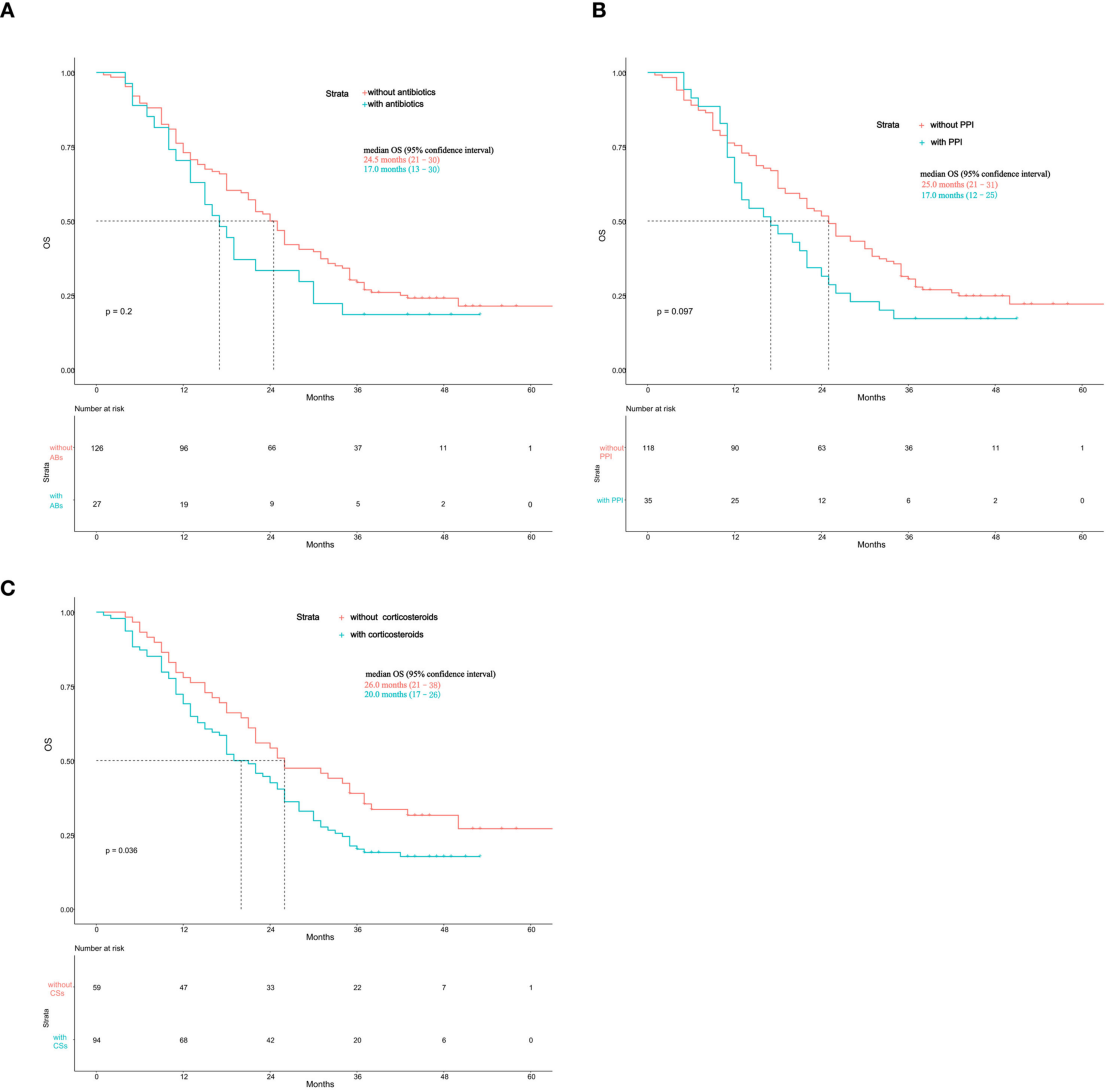
**(A)** Kaplan-Meier overall survival curves for patients with or without antibiotic use. ABs, antibiotics. **(B)** Kaplan-Meier overall survival curves for patients with or without proton pump inhibitor use. PPI, proton pump inhibitor. **(C)** Kaplan-Meier overall survival curves for patients with or without corticosteroids use. CSs, corticosteroids.

Kaplan-Meier analyses were performed to evaluate the impact of concomitant medication use on OS among patients with advanced NSCLC treated with ICIs. Patients who received antibiotics showed a trend toward shorter overall survival compared with those who did not (**Figure 3A**); however, the difference was not statistically significant (median OS: 24.5 months vs. 17.0 months, *p* = 0.2). Similarly, use of proton pump inhibitors was associated with a numerically shorter OS than non−use, but the difference also did not reach statistical significance (median OS: 25.0 months vs. 17.0 months, *p* = 0.097) (**Figure 3B**). In contrast, patients who received corticosteroids had significantly shorter overall survival than those who did not (median OS: 26.0 months vs. 20.0 months, *p* = 0.036) (**Figure 3C**). Overall, these results suggest that concomitant corticosteroid use is associated with inferior survival outcomes, while antibiotic and proton pump inhibitors use showed a non−significant trend toward reduced overall survival in patients treated with ICIs.

### 3.5 Comparison of survival outcomes between locally and internationally developed ICIs, and among different PD-1 inhibitors

**Figure 4:**
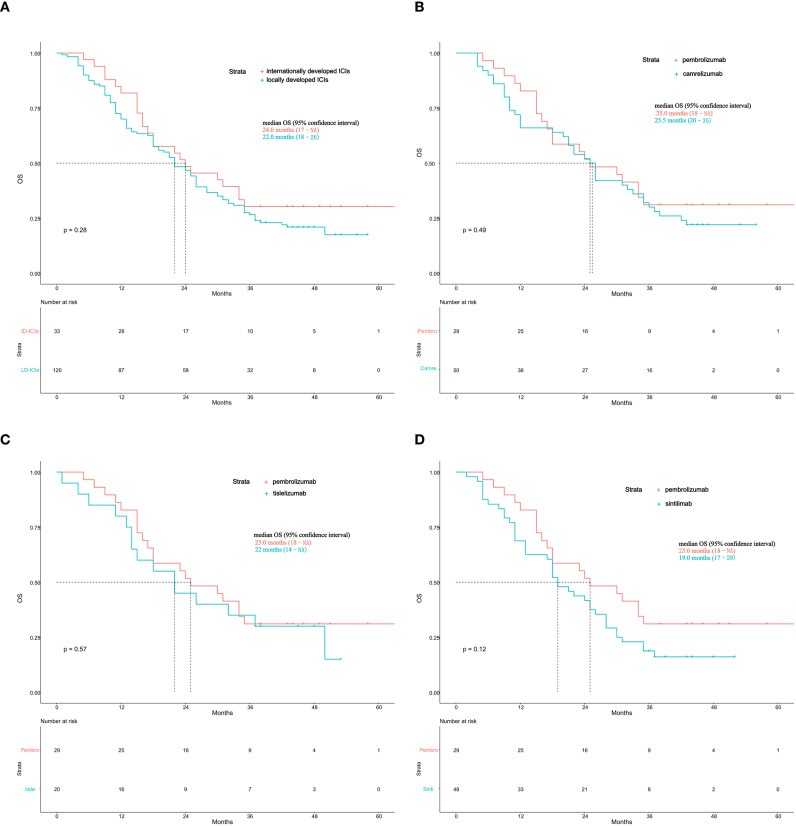
**(A)** Kaplan-Meier curves for overall survival comparing locally developed and internationally developed ICIs. ID-ICIs, internationally developed ICIs; LD-ICIs, locally developed ICIs. **(B)** Kaplan-Meier curves for overall survival comparing pembrolizumab and camrelizumab. Pembro, pembrolizumab; Camre, camrelizumab. **(C)** Kaplan-Meier curves for overall survival comparing pembrolizumab and tislelizumab. Pembro, pembrolizumab; Tisle, tislelizumab. **(D)** Kaplan-Meier curves for overall survival comparing pembrolizumab and sintilimab. Pembro, pembrolizumab; Sinti, sintilimab.

To explore potential differences in survival outcomes between locally and internationally developed ICIs, we conducted comparative survival analyses of the two groups. Baseline clinical characteristics were balanced between patients treated with locally developed and internationally developed ICIs, with no significant differences observed in sex, age, body mass index, comorbidities, tumor stage, histology, or PD−L1 expression levels (all *p* > 0.05; **Table 3**). In the survival analysis, there was no statistically significant difference in OS between locally developed and internationally developed ICIs (median OS 22.0 months [95% CI 18-26] vs 24.0 months [95% CI 17-NA]; *p* = 0.28; **Figure 4A**).

**Table 3:** Baseline characteristics of patients according to the origin of ICIs (locally developed vs. internationally developed).

Characteristics	[ALL] N=153	Internationally developed ICIs N = 33	Locally developed ICIs N=120	*P* value
Sex, n (%)				0.954
Female	25 (16.3%)	6 (18.2%)	19 (15.8%)	
Male	128 (83.7%)	27 (81.8%)	101 (84.2%)	
Age (years), n (%)				0.217
<60	49 (32.0%)	14 (42.4%)	35 (29.2%)	
≥60	104 (68.0%)	19 (57.6%)	85 (70.8%)	
BMI, n (%)				0.339
<18.5	13 (8.50%)	2 (6.06%)	11 (9.17%)	
18.5-24.9	96 (62.7%)	18 (54.5%)	78 (65.0%)	
≥25	44 (28.8%)	13 (39.4%)	31 (25.8%)	
Nutrition Score, n (%)				0.779
<3	132 (86.3%)	28 (84.8%)	104 (86.7%)	
≥3	21 (13.7%)	5 (15.2%)	16 (13.3%)	
Smoking, n (%)				0.925
No	38 (24.8%)	9 (27.3%)	29 (24.2%)	
Yes	115 (75.2%)	24 (72.7%)	91 (75.8%)	
Drinking, n (%)				0.379
No	89 (58.2%)	18 (54.5%)	71 (59.2%)	
Yes	64 (41.8%)	15 (45.5%)	49 (40.8%)	
Diabetes comorbidity, n (%)				0.368
No	136 (88.9%)	31 (93.9%)	105 (87.5%)	
Yes	17 (11.1%)	2 (6.06%)	15 (12.5%)	
Cardiovascular diseases comorbidity, n (%)				1.000
No	116 (75.8%)	25 (75.8%)	91 (75.8%)	
Yes	37 (24.2%)	8 (24.2%)	29 (24.2%)	
Lung Disease Comorbidity, n (%)				0.440
No	115 (75.2%)	27 (81.8%)	88 (73.3%)	
Yes	38 (24.8%)	6 (18.2%)	32 (26.7%)	
T stage, n (%)				0.457
1-2	77 (50.3%)	19 (57.6%)	58 (48.3%)	
3-4	76 (49.7%)	14 (42.4%)	62 (51.7%)	
N stage, n (%)				1.000
0-1	21 (13.7%)	4 (12.1%)	17 (14.2%)	
2-3	132 (86.3%)	29 (87.9%)	103 (85.8%)	
Histological type, n (%)				0.428
Non-squamous	96 (62.7%)	24 (72.7%)	72 (60.0%)	
Squamous	54 (35.3%)	9 (27.3%)	45 (37.5%)	
Unknown	3 (1.96%)	0 (0.00%)	3 (2.50%)	
Liver metastasis, n (%)				0.251
No	132 (86.3%)	31 (93.9%)	101 (84.2%)	
Yes	21 (13.7%)	2 (6.06%)	19 (15.8%)	
Bone metastasis, n (%)				0.916
No	87 (56.9%)	18 (54.5%)	69 (57.5%)	
Yes	66 (43.1%)	15 (45.5%)	51 (42.5%)	
Brain metastasis, n (%)				0.485
No	116 (75.8%)	23 (69.7%)	93 (77.5%)	
Yes	37 (24.2%)	10 (30.3%)	27 (22.5%)	
PD-L1 expression, n (%)				0.962
PD-L1 <1%	20 (13.1%)	4 (12.1%)	16 (13.3%)	
PD-L1≥1%	70 (45.8%)	16 (48.5%)	54 (45.0%)	
Unknown	63 (41.2%)	13 (39.4%)	50 (41.7%)	
Antibiotic exposure, n (%)				0.168
No	126 (82.4%)	24 (72.7%)	102 (85.0%)	
Yes	27 (17.6%)	9 (27.3%)	18 (15.0%)	
PPI exposure, n (%)				0.361
No	118 (77.1%)	23 (69.7%)	95 (79.2%)	
Yes	35 (22.9%)	10 (30.3%)	25 (20.8%)	
Corticosteroid exposure, n (%)				1.000
No	59 (38.6%)	13 (39.4%)	46 (38.3%)	
Yes	94 (61.4%)	20 (60.6%)	74 (61.7%)	
Dead, n (%)				0.361
No	35 (22.9%)	10 (30.3%)	25 (20.8%)	
Yes	118 (77.1%)	23 (69.7%)	95 (79.2%)	

We further conducted comparative survival analyses between pembrolizumab—the most widely used imported PD−1 inhibitor—and each of the three locally developed PD−1 inhibitors: camrelizumab, tislelizumab, and sintilimab. Baseline demographic and clinical characteristics were generally comparable across the four treatment groups. However, histological type differed significantly among the groups (*p* = 0.005; **Table 4**), with a higher proportion of squamous NSCLC in the tislelizumab group. Consistent with the overall comparison, the Kaplan-Meier curves showed no statistically significant differences in overall survival between pembrolizumab and camrelizumab (median OS 25.5 vs 25.0 months; *p* = 0.49; **Figure 4B**), pembrolizumab and tislelizumab (25.0 vs 22.0 months; *p* = 0.57; **Figure 4C**), or pembrolizumab and sintilimab (25.0 vs 19.0 months; *p* = 0.12; **Figure 4D**). Taken together, these findings showed no statistically significant difference in overall survival between locally developed ICIs and pembrolizumab in this real-world retrospective cohort. However, this comparison should be interpreted cautiously because the internationally developed ICI group was relatively small, limiting statistical power for comparative inference.

**Table 4:** Baseline characteristics of patients according to the type of PD−1 inhibitor (pembrolizumab, camrelizumab, tislelizumab, and sintilimab).

Characteristics	[ALL] N = 147	Pembrolizumab N = 29	Camrelizumab N = 50	Tislelizumab N = 20	Sintilimab N = 48	*P* value
Sex, n (%)						0.575
Female	22 (15.0%)	4 (13.8%)	8 (16.0%)	1 (5.00%)	9 (18.8%)	
Male	125 (85.0%)	25 (86.2%)	42 (84.0%)	19 (95.0%)	39 (81.2%)	
Age (years), n (%)						0.270
<60	47 (32.0%)	13 (44.8%)	14 (28.0%)	4 (20.0%)	16 (33.3%)	
≥60	100 (68.0%)	16 (55.2%)	36 (72.0%)	16 (80.0%)	32 (66.7%)	
BMI, n (%)						0.483
<18.5	13 (8.84%)	2 (6.90%)	2 (4.00%)	3 (15.0%)	6 (12.5%)	
18.5-24.9	95 (64.6%)	17 (58.6%)	35 (70.0%)	14 (70.0%)	29 (60.4%)	
>25	39 (26.5%)	10 (34.5%)	13 (26.0%)	3 (15.0%)	13 (27.1%)	
Nutrition Score, n (%)						0.161
<3	126 (85.7%)	24 (82.8%)	47 (94.0%)	17 (85.0%)	38 (79.2%)	
≥3	21 (14.3%)	5 (17.2%)	3 (6.00%)	3 (15.0%)	10 (20.8%)	
Smoking, n (%)						0.970
No	35 (23.8%)	7 (24.1%)	13 (26.0%)	4 (20.0%)	11 (22.9%)	
Yes	112 (76.2%)	22 (75.9%)	37 (74.0%)	16 (80.0%)	37 (77.1%)	
Drinking, n (%)						0.186
No	86 (58.5%)	16 (55.2%)	26 (52.0%)	16 (80.0%)	28 (58.3%)	
Yes	61 (41.5%)	13 (44.8%)	24 (48.0%)	4 (20.0%)	20 (41.7%)	
Diabetes comorbidity, n (%)						0.553
No	131 (89.1%)	27 (93.1%)	45 (90.0%)	16 (80.0%)	43 (89.6%)	
Yes	16 (10.9%)	2 (6.90%)	5 (10.0%)	4 (20.0%)	5 (10.4%)	
Cardiovascular diseases comorbidity, n (%)						1.000
No	112 (76.2%)	22 (75.9%)	38 (76.0%)	15 (75.0%)	37 (77.1%)	
Yes	35 (23.8%)	7 (24.1%)	12 (24.0%)	5 (25.0%)	11 (22.9%)	
Lung Disease Comorbidity, n (%)						0.381
No	111 (75.5%)	24 (82.8%)	40 (80.0%)	13 (65.0%)	34 (70.8%)	
Yes	36 (24.5%)	5 (17.2%)	10 (20.0%)	7 (35.0%)	14 (29.2%)	
T stage, n (%)						0.368
1-2	73 (49.7%)	16 (55.2%)	28 (56.0%)	7 (35.0%)	22 (45.8%)	
3-4	74 (50.3%)	13 (44.8%)	22 (44.0%)	13 (65.0%)	26 (54.2%)	
N stage, n (%)						0.142
0-1	20 (13.6%)	4 (13.8%)	6 (12.0%)	6 (30.0%)	4 (8.33%)	
2-3	127 (86.4%)	25 (86.2%)	44 (88.0%)	14 (70.0%)	44 (91.7%)	
Histological type, n (%)						0.005
Non-squamous	91 (61.9%)	21 (72.4%)	37 (74.0%)	6 (30.0%)	27 (56.2%)	
Squamous	53 (36.1%)	8 (27.6%)	11 (22.0%)	14 (70.0%)	20 (41.7%)	
Unknown	3 (2.04%)	0 (0.00%)	2 (4.00%)	0 (0.00%)	1 (2.08%)	
Liver metastasis, n (%)						0.253
No	126 (85.7%)	27 (93.1%)	44 (88.0%)	18 (90.0%)	37 (77.1%)	
Yes	21 (14.3%)	2 (6.90%)	6 (12.0%)	2 (10.0%)	11 (22.9%)	
Bone metastasis, n (%)						0.754
No	83 (56.5%)	16 (55.2%)	27 (54.0%)	10 (50.0%)	30 (62.5%)	
Yes	64 (43.5%)	13 (44.8%)	23 (46.0%)	10 (50.0%)	18 (37.5%)	
Brain metastasis, n (%)						0.264
No	113 (76.9%)	21 (72.4%)	35 (70.0%)	18 (90.0%)	39 (81.2%)	
Yes	34 (23.1%)	8 (27.6%)	15 (30.0%)	2 (10.0%)	9 (18.8%)	
PD-L1 expression, n (%)						0.280
PD-L1 <1%	19 (12.9%)	3 (10.3%)	8 (16.0%)	5 (25.0%)	3 (6.25%)	
PD-L1≥1%	70 (47.6%)	16 (55.2%)	23 (46.0%)	10 (50.0%)	21 (43.8%)	
Unknown	58 (39.5%)	10 (34.5%)	19 (38.0%)	5 (25.0%)	24 (50.0%)	
Antibiotic exposure, n (%)						0.075
No	120 (81.6%)	20 (69.0%)	45 (90.0%)	18 (90.0%)	37 (77.1%)	
Yes	27 (18.4%)	9 (31.0%)	5 (10.0%)	2 (10.0%)	11 (22.9%)	
PPI exposure, n (%)						0.465
No	113 (76.9%)	20 (69.0%)	41 (82.0%)	14 (70.0%)	38 (79.2%)	
Yes	34 (23.1%)	9 (31.0%)	9 (18.0%)	6 (30.0%)	10 (20.8%)	
Corticosteroid exposure, n (%)						0.696
No	55 (37.4%)	10 (34.5%)	18 (36.0%)	6 (30.0%)	21 (43.8%)	
Yes	92 (62.6%)	19 (65.5%)	32 (64.0%)	14 (70.0%)	27 (56.2%)	
Dead, n (%)						0.510
No	33 (22.4%)	9 (31.0%)	11 (22.0%)	5 (25.0%)	8 (16.7%)	
Yes	114 (77.6%)	20 (69.0%)	39 (78.0%)	15 (75.0%)	40 (83.3%)	

## 4 Discussion

In recent years, ICIs have emerged as one of the most widely adopted immunotherapeutic approaches in oncology worldwide. Multiple agents targeting PD-1, PD-L1, cytotoxic T-lymphocyte-associated protein-4 (CTLA-4) and lymphocyte activation gene-3 (LAG-3) have received regulatory approval for clinical use across various stages of diverse solid malignancies.

The widespread adoption of ICIs has markedly transformed the therapeutic landscape of solid tumors, particularly for patients with driver mutation–negative advanced NSCLC. Evidence from multiple randomized controlled trials has consistently demonstrated the clinical benefit of ICIs. In pivotal phase III studies such as KEYNOTE−189 (11) and KEYNOTE−407 (12), pembrolizumab combined with chemotherapy significantly improved median overall survival compared with chemotherapy alone (22.0 vs 10.6 months, *p* < 0.001; and 17.1 vs 11.6 months, *p* = 0.0018, respectively). These findings highlight the durable efficacy of PD−1 inhibitor regimens, which have become a cornerstone of first−line systemic therapy for advanced NSCLC.

In parallel with global phase III studies, several randomized trials conducted in China have also demonstrated significant survival benefits with domestically developed PD-1 inhibitors in advanced NSCLC, including CameL, CameL-Sq, ORIENT-11, and RATIONALE-307 (13–20). These studies further support the role of PD-1 blockade across different histologic subtypes and clinical settings in the Chinese population.

Currently, the combination of ICIs with chemotherapy represents the standard first-line treatment for patients with advanced NSCLC lacking driver gene alterations. However, real-world populations receiving immuno-oncology regimens often exhibit considerable clinical heterogeneity compared with the highly selected cohorts enrolled in pivotal clinical trials. Consequently, real-world evidence has become increasingly important for assessing the effectiveness of ICIs-based therapies across diverse patient populations (21).

In the present study, 401 patients with driver mutation-negative advanced NSCLC were included to compare first-line outcomes between chemotherapy alone and ICIs ± chemotherapy. The results demonstrated a significantly longer OS in the immunotherapy cohort, with a median OS of 22.0 months versus 14.0 months (*p* < 0.001), confirming substantial survival benefits with ICIs-based regimens over conventional chemotherapy. Importantly, this survival advantage remained significant in the propensity score-matched cohort, further supporting the robustness of the primary findings after balancing baseline characteristics between the two treatment groups.

Our findings should also be interpreted in the context of pivotal randomized trials of first-line immunotherapy for advanced NSCLC. In KEYNOTE-189 (11) and KEYNOTE-407 (12), pembrolizumab plus chemotherapy significantly improved overall survival compared with chemotherapy alone in patients with non-squamous and squamous NSCLC, respectively. Similarly, IMpower130 (10) demonstrated a survival benefit with atezolizumab plus chemotherapy in metastatic non-squamous NSCLC. The consistency in the direction of benefit between these landmark trials and our real-world analysis supports the clinical effectiveness of first-line ICIs-based therapy in routine practice.

At the same time, our study differs from these randomized trials in both design and clinical context. Unlike highly selected trial populations, our cohort reflects routine clinical practice and includes a broader and more heterogeneous population, which may better capture treatment effectiveness in daily oncology care1. Therefore, our findings should be viewed as complementary real-world evidence rather than a direct cross-trial comparison with KEYNOTE or IMpower studies.

In addition to the overall survival advantage associated with ICIs-based therapy, our multivariable Cox regression analysis identified T stage, bone metastasis, and first-line treatment regimen as independent prognostic factors for OS. Specifically, more advanced T stage and the presence of bone metastasis were independently associated with poorer survival, whereas receipt of first-line immunotherapy was associated with improved OS. These findings are clinically plausible. Advanced T stage usually reflects greater local tumor burden and more aggressive disease behavior, both of which may contribute to inferior outcomes. Bone metastasis is a well-recognized adverse prognostic factor in advanced NSCLC and may indicate more disseminated disease, increased symptom burden, and a poorer systemic condition. Importantly, even after adjustment for these prognostic variables, first-line ICIs-based treatment remained significantly associated with better survival, further supporting the robustness of the survival benefit observed in our real-world cohort.

Our study supports the superior effectiveness of immunotherapy over chemotherapy in this real-world cohort. Exploratory subgroup analyses suggested potential heterogeneity in treatment effect according to BMI, pre-existing pulmonary disease, smoking status, and T stage; however, these findings should be interpreted cautiously given the retrospective design and the limited sample sizes within subgroups.

In the exploratory subgroup analyses, BMI was associated with a possible difference in the relative survival benefit of ICIs-based therapy. Both overweight/obese and underweight patients appeared to derive greater benefit than those with normal BMI. For overweight or obese patients, this observation is broadly consistent with previous reports describing an “obesity paradox” in the setting of immune checkpoint blockade (22, 23). However, the biological mechanisms underlying this association remain incompletely understood. Our finding of a possible benefit in underweight patients contrasts with most previous studies, which have generally linked low BMI or pretreatment weight loss to poorer outcomes in patients receiving immunotherapy (24, 25). Therefore, this result should be interpreted with caution, particularly given the small number of underweight patients in our cohort and the possibility of residual confounding. Although differences in host metabolic status, immune function, or chemotherapy tolerance may have contributed to this pattern, these explanations cannot be tested in the present retrospective dataset. Accordingly, the BMI-related subgroup finding should be regarded as hypothesis-generating rather than conclusive.

Pre-existing pulmonary disease was also associated with a possible difference in survival benefit in the exploratory subgroup analysis. This finding requires cautious interpretation and external validation. Previous studies have suggested that chronic pulmonary comorbidity may be associated with changes in tumor immunogenicity, PD-L1 expression, tumor mutational burden, and immune-cell infiltration (26–29). However, these mechanisms were not evaluated in our study and remain speculative in the present context.

Smoking status was likewise associated with a possible difference in treatment effect in the exploratory analysis, with current or former smokers appearing to derive greater relative benefit from ICIs-based therapy than never-smokers. This observation is biologically plausible in light of prior evidence linking tobacco exposure to increased tumor mutational burden and altered tumor immunogenicity (30, 31). However, given the retrospective design and limited power for interaction analyses, no firm conclusions regarding effect modification can be drawn from the current study.

Patients with earlier T-stage tumors also appeared to derive greater relative benefit from ICIs-based therapy in the exploratory subgroup analysis. This finding may be consistent with previous observations that lower tumor burden is associated with improved outcomes after immune checkpoint inhibition (32–34). However, in the present study, T stage served only as a rough clinical surrogate and did not capture detailed tumor burden. Accordingly, this finding should be interpreted cautiously and considered hypothesis-generating.

The efficacy of immune checkpoint inhibitors is not solely determined by tumor-intrinsic factors but is profoundly influenced by host-related variables. Among these, concomitant medications that modulate the host immune system or the gut microbiome have garnered significant attention. A growing body of evidence has suggested that the use of antibiotics, proton pump inhibitors, and corticosteroids can exert negative impacts on ICIs outcomes, potentially through mechanisms such as microbiota dysbiosis or direct immunosuppression (35–41). Considering their potential clinical impact, we further assessed the association between concomitant antibiotics, proton pump inhibitors, and corticosteroid use and survival outcomes in our ICIs−treated cohort.

In our cohort, concomitant corticosteroid use was significantly associated with shorter overall survival in patients treated with immune checkpoint inhibitors, whereas antibiotic and proton pump inhibitor use showed a non−significant trend toward reduced survival. These findings are generally consistent with previous studies suggesting that host−related medications may influence the efficacy of immunotherapy.

The detrimental impact of corticosteroids has been well established. Corticosteroids can suppress T−cell activation, reduce cytokine production, and promote an immunosuppressive tumor microenvironment, thereby diminishing the therapeutic effects of ICIs. Although corticosteroids are often clinically necessary for symptom control or management of immune−related adverse events, their baseline or early use during immunotherapy has been associated with poorer survival outcomes (39, 42). Our findings further support these observations.

The effects of antibiotics and proton pump inhibitors remain inconclusive. Both drug classes may alter the gut microbiota composition and reduce microbial diversity, potentially interfering with immune activation. Some studies have reported poorer outcomes in patients exposed to these medications, whereas others found no significant association (43–46). In our cohort, no statistically significant differences were observed, which may be attributable to sample size limitations or variability in drug exposure. Further studies are warranted to clarify their clinical relevance.

Taken together, these findings showed no statistically significant difference in overall survival between locally developed ICIs and pembrolizumab in this real-world retrospective cohort. However, this comparison should be interpreted cautiously because the internationally developed ICIs group was relatively small, limiting statistical power for comparative inference. A previous systematic review and indirect comparison also suggested that survival outcomes with tislelizumab plus chemotherapy and pembrolizumab plus chemotherapy may be broadly similar, although cross-trial comparisons have important inherent limitations (47). Because all PD-1 inhibitors target the same PD-1/PD-L1 axis, some overlap in clinical activity may be expected. Nevertheless, our study was not designed or powered to determine equivalence among agents, and the present findings should be viewed as descriptive rather than definitive comparative evidence. Beyond efficacy considerations, the broader availability of locally developed ICIs may improve access to immunotherapy in routine clinical practice and may have practical implications for treatment affordability in China.

Nevertheless, several limitations should be acknowledged. First, residual confounding cannot be excluded because of the retrospective, non-randomized design, despite multivariable adjustment. Several potentially important prognostic variables, including ECOG performance status, detailed tumor burden, comorbidity burden, and treatment era effects, were not consistently available for adjustment. Second, treatment selection may have been influenced by physician preference and patient socioeconomic factors, introducing potential selection bias. Third, PD-L1 expression was unavailable in a substantial proportion of patients, which may have affected treatment allocation and the adequacy of confounding control. Fourth, the ICIs-based group included both ICIs monotherapy and chemoimmunotherapy, introducing potential treatment heterogeneity, although most patients received combination therapy. Fifth, this was a single-center study, which may limit generalizability. In addition, corticosteroid exposure was defined within a prespecified time window, whereas detailed data on indication, duration, and cumulative dose were not uniformly available. Some subgroup analyses were exploratory and based on relatively small sample sizes, limiting statistical power and increasing the possibility of chance findings. The biological interpretations of these subgroup patterns also remain speculative, as molecular and immunologic correlates were not available in this retrospective dataset. Moreover, the comparison between locally developed and internationally developed ICIs was limited by substantial sample-size imbalance, particularly the small number of patients in the internationally developed ICIs group, precluding any definitive conclusion regarding equivalence or comparative effectiveness. Therefore, these findings should be considered hypothesis-generating and warrant validation in larger prospective multicenter studies with more complete clinical and biomarker data.

## 5 Conclusion

In this real−world study of patients with advanced non−small−cell lung cancer, first−line immunotherapy achieved significantly better survival outcomes than chemotherapy alone. The treatment benefit was generally consistent across clinical subgroups, although exploratory analyses suggested possible heterogeneity in selected patient subsets that requires further validation. No statistically significant difference in overall survival was observed between locally developed PD-1 inhibitors and pembrolizumab in this cohort; however, this finding should be interpreted cautiously given the limited sample size and requires confirmation in larger comparative studies.

## Data Availability

The raw data supporting the conclusions of this article will be made available by the authors, without undue reservation.

## References

[1] Filho AM Laversanne M Ferlay J Colombet M Piñeros M Znaor A . The GLOBOCAN 2022 cancer estimates: Data sources, methods, and a snapshot of the cancer burden worldwide. Int J Cancer. (2025) 156:16–1346. doi: 10.1002/ijc.35278. PMID: 39688499

[2] Xia C Dong X Li H Cao M Sun D He S . Cancer statistics in China and United States, 2022: Profiles, trends, and determinants. Chin Med J (Engl). (2022) 135:584–90. doi: 10.1097/CM9.0000000000002108. PMID: 35143424 PMC8920425

[3] Sun C Mezzadra R Schumacher TN . Regulation and function of the PD-L1 checkpoint. Immunity. (2018) 48:434–52. doi: 10.1016/j.immuni.2018.03.014. PMID: 29562194 PMC7116507

[4] Ribas A Wolchok JD . Cancer immunotherapy using checkpoint blockade. Science. (2018) 359:1350–5. doi: 10.1126/science.aar4060. PMID: 29567705 PMC7391259

[5] Wei SC Duffy CR Allison JP . Fundamental mechanisms of immune checkpoint blockade therapy. Cancer Discov. (2018) 8:1069–86. doi: 10.1158/2159-8290.CD-18-0367. PMID: 30115704

[6] Reck M Rodríguez-Abreu D Robinson AG Hui R Csőszi T Fülöp A . Five-year outcomes with pembrolizumab versus chemotherapy for metastatic non-small-cell lung cancer with PD-L1 tumor proportion score ≥50. J Clin Oncol. (2021) 39:2339–49. doi: 10.1200/JCO.21.00174. PMID: 33872070 PMC8280089

[7] Reck M Rodríguez-Abreu D Robinson AG Hui R Csőszi T Fülöp A . Pembrolizumab versus chemotherapy for PD-L1-positive non-small-cell lung cancer. N Engl J Med. (2016) 375:1823–33. doi: 10.1056/NEJMoa1606774. PMID: 27718847

[8] de Castro G Kudaba I Wu YL Lopes G Kowalski DM Turna HZ . Five-year outcomes with pembrolizumab versus chemotherapy as first-line therapy in patients with non-small-cell lung cancer and programmed death ligand-1 tumor proportion score ≥ 1% in the KEYNOTE-042 study. J Clin Oncol. (2023) 41:1986–91. doi: 10.1200/JCO.21.02885. PMID: 36306479 PMC10082298

[9] Mok TSK Wu YL Kudaba I Kowalski DM Cho BC Turna HZ . Pembrolizumab versus chemotherapy for previously untreated, PD-L1-expressing, locally advanced or metastatic non-small-cell lung cancer (KEYNOTE-042): a randomised, open-label, controlled, phase 3 trial. Lancet. (2019) 393:1819–30. doi: 10.1016/S0140-6736(18)32409-7. PMID: 30955977

[10] West H McCleod M Hussein M Morabito A Rittmeyer A Conter HJ . Atezolizumab in combination with carboplatin plus nab-paclitaxel chemotherapy compared with chemotherapy alone as first-line treatment for metastatic non-squamous non-small-cell lung cancer (IMpower130): a multicentre, randomised, open-label, phase 3 trial. Lancet Oncol. (2019) 20:924–37. doi: 10.1016/S1470-2045(19)30167-6. PMID: 31122901

[11] Rodríguez-Abreu D Powell SF Hochmair MJ Gadgeel S Esteban E Felip E . Pemetrexed plus platinum with or without pembrolizumab in patients with previously untreated metastatic nonsquamous NSCLC: protocol-specified final analysis from KEYNOTE-189. Ann Oncol. (2021) 32:881–95. doi: 10.1016/j.annonc.2021.04.008. PMID: 33894335

[12] Paz-Ares L Vicente D Tafreshi A Robinson A Soto Parra H Mazières J . A randomized, placebo-controlled trial of pembrolizumab plus chemotherapy in patients with metastatic squamous NSCLC: protocol-specified final analysis of KEYNOTE-407. J Thorac Oncol. (2020) 15:1657–69. doi: 10.1016/j.jtho.2020.06.015. PMID: 32599071

[13] Zhou C Chen G Huang Y Zhou J Lin L Feng J . Camrelizumab plus carboplatin and pemetrexed as first-line treatment for advanced nonsquamous NSCLC: extended follow-up of CameL phase 3 trial. J Thorac Oncol. (2023) 18:628–39. doi: 10.1016/j.jtho.2022.12.017. PMID: 36646210

[14] Zhou C Chen G Huang Y Zhou J Lin L Feng J . Camrelizumab plus carboplatin and pemetrexed as first-line therapy for advanced non-squamous non-small-cell lung cancer: 5-year outcomes of the CameL randomized phase 3 study. J Immunother Cancer. (2024) 12:e009240. doi: 10.1136/jitc-2024-009240. PMID: 39608979 PMC11603811

[15] Yang Y Wang Z Fang J Yu Q Han B Cang S . Efficacy and safety of sintilimab plus pemetrexed and platinum as first-line treatment for locally advanced or metastatic nonsquamous NSCLC: a randomized, double-blind, phase 3 study (Oncology pRogram by InnovENT anti-PD-1-11). J Thorac Oncol. (2020) 15:1636–46. doi: 10.1016/j.jtho.2020.07.014. PMID: 32781263

[16] Zhang L Wang Z Fang J Yu Q Han B Cang S . Final overall survival data of sintilimab plus pemetrexed and platinum as first-line treatment for locally advanced or metastatic nonsquamous NSCLC in the Phase 3 ORIENT-11 study. Lung Cancer. (2022) 171:56–60. doi: 10.1016/j.lungcan.2022.07.013. PMID: 35917647

[17] Liu T He J Wang Y Yang Y Zhang L Shi M . Health-related quality of life and symptoms in patients with previously untreated, locally advanced or metastatic non-squamous non-small cell lung cancer treated with sintilimab or placebo plus pemetrexed and platinum (ORIENT-11): A randomized, double-blind, phase 3 trial. Lung Cancer. (2025) 200:108108. doi: 10.1016/j.lungcan.2025.108108. PMID: 39884222

[18] Ren S Chen J Xu X Jiang T Cheng Y Chen G . Camrelizumab plus carboplatin and paclitaxel as first-line treatment for advanced squamous NSCLC (CameL-Sq): A Phase 3 Trial. J Thorac Oncol. (2022) 17:544–57. doi: 10.1016/j.jtho.2021.11.018. PMID: 34923163

[19] Zhou C Huang D Fan Y Yu X Liu Y Shu Y . Tislelizumab versus docetaxel in patients with previously treated advanced NSCLC (RATIONALE-303): A phase 3, open-label, randomized controlled trial. J Thorac Oncol. (2023) 18:93–105. doi: 10.1016/j.jtho.2022.09.217. PMID: 36184068

[20] Wang J Lu S Yu X Hu Y Zhao J Sun M . Tislelizumab plus chemotherapy versus chemotherapy alone as first-line treatment for advanced squamous non-small-cell lung cancer: final analysis of the randomized, phase III RATIONALE-307 trial. ESMO Open. (2024) 9:103727. doi: 10.1016/j.esmoop.2024.103727. PMID: 39461775 PMC11549530

[21] Blonde L Khunti K Harris SB Meizinger C Skolnik NS . Interpretation and impact of real-world clinical data for the practicing clinician. Adv Ther. (2018) 35:1763–74. doi: 10.1007/s12325-018-0805-y. PMID: 30357570 PMC6223979

[22] Alden SL Charmsaz S Li HL Tsai HL Danilova L Munjal K . Pan-tumor analysis to investigate the obesity paradox in immune checkpoint blockade. J Immunother Cancer. (2025) 13:e009734. doi: 10.1136/jitc-2024-009734. PMID: 39832896 PMC11748946

[23] Mastrolonardo EV Llerena P De Ravin E Nunes K Kaki PC Bridgham KM . Improved survival with elevated BMI following immune checkpoint inhibition across various solid tumor cancer types. Cancer. (2025) 131:e35799. doi: 10.1002/cncr.35799. PMID: 40069917 PMC11897419

[24] Ichihara E Harada D Inoue K Sato K Hosokawa S Kishino D . The impact of body mass index on the efficacy of anti-PD-1/PD-L1 antibodies in patients with non-small cell lung cancer. Lung Cancer. (2020) 139:140–5. doi: 10.1016/j.lungcan.2019.11.011. PMID: 31786476

[25] Jin J Visina J Burns TF Diergaarde B Stabile LP . Male sex and pretreatment weight loss are associated with poor outcome in patients with advanced non-small cell lung cancer treated with immunotherapy: a retrospective study. Sci Rep. (2023) 13:17047. doi: 10.1038/s41598-023-43866-5. PMID: 37813923 PMC10562448

[26] Biton J Ouakrim H Dechartres A Alifano M Mansuet-Lupo A Si H . Impaired tumor-infiltrating T cells in patients with chronic obstructive pulmonary disease impact lung cancer response to PD-1 blockade. Am J Respir Crit Care Med. (2018) 198:928–40. doi: 10.1164/rccm.201706-1110OC. PMID: 29518341

[27] Riondino S Rosenfeld R Formica V Morelli C Parisi G Torino F . Effectiveness of immunotherapy in non-small cell lung cancer patients with a diagnosis of COPD: is this a hidden prognosticator for survival and a risk factor for immune-related adverse events? Cancers (Basel). (2024) 16:1251. doi: 10.3390/cancers16071251. PMID: 38610929 PMC11011072

[28] Lycan TW Norton DL Ohar JA . COPD and immune checkpoint inhibitors for cancer: a literature review. Int J Chron Obstruct Pulmon Dis. (2024) 19:2689–703. doi: 10.2147/COPD.S490252. PMID: 39677829 PMC11639883

[29] Zhang Q Feng X Hu W Li C Sun D Peng Z . Chronic obstructive pulmonary disease alters the genetic landscape and tumor immune microenvironment in lung cancer patients. Front Oncol. (2023) 13:1169874. doi: 10.3389/fonc.2023.1169874. PMID: 37388220 PMC10301745

[30] Rizvi NA Hellmann MD Snyder A Kvistborg P Makarov V Havel JJ . Cancer immunology. Mutational landscape determines sensitivity to PD-1 blockade in non-small cell lung cancer. Science. (2015) 348:124–8. doi: 10.1126/science.aaa1348. PMID: 25765070 PMC4993154

[31] Wang X Ricciuti B Alessi JV Nguyen T Awad MM Lin X . Smoking history as a potential predictor of immune checkpoint inhibitor efficacy in metastatic non-small cell lung cancer. J Natl Cancer Inst. (2021) 113:1761–9. doi: 10.1093/jnci/djab116. PMID: 34115098 PMC8634315

[32] Katsurada M Nagano T Tachihara M Kiriu T Furukawa K Koyama K . Baseline tumor size as a predictive and prognostic factor of immune checkpoint inhibitor therapy for non-small cell lung cancer. Anticancer Res. (2019) 39:815–25. doi: 10.21873/anticanres.13180. PMID: 30711962

[33] Wen Z Sun H Zhang Z Zheng Y Zheng S Bin J . High baseline tumor burden-associated macrophages promote an immunosuppressive microenvironment and reduce the efficacy of immune checkpoint inhibitors through the IGFBP2-STAT3-PD-L1 pathway. Cancer Commun (Lond). (2023) 43:562–81. doi: 10.1002/cac2.12420. PMID: 37031362 PMC10174084

[34] Lazzari C Spagnolo CC Ciappina G Di Pietro M Squeri A Passalacqua MI . Immunotherapy in early-stage non-small cell lung cancer (NSCLC): current evidence and perspectives. Curr Oncol. (2023) 30:3684–96. doi: 10.3390/curroncol30040280. PMID: 37185393 PMC10136903

[35] Alkan Şen G Şentürk Öztaş N Değerli E Can G Turna H Özgüroğlu M . Effect of antibiotic treatment on immune checkpoint inhibitors efficacy in patients with advanced non-small cell lung cancer. Lung Cancer. (2023) 184:107347. doi: 10.1016/j.lungcan.2023.107347. PMID: 37597304

[36] Zhong J Xiong D Liu Y Yuan S . Association of antibiotic exposure with survival in patients with extensive-stage small cell lung cancer receiving immune checkpoint inhibitor therapy. Thorac Cancer. (2024) 15:152–62. doi: 10.1111/1759-7714.15172. PMID: 38010059 PMC10788467

[37] Qin BD Jiao XD Zhou XC Shi B Wang J Liu K . Effects of concomitant proton pump inhibitor use on immune checkpoint inhibitor efficacy among patients with advanced cancer. Oncoimmunology. (2021) 10:1929727. doi: 10.1080/2162402X.2021.1929727. PMID: 34350061 PMC8296970

[38] Hu DH Wong WC Zhou JX Luo J Cai SW Zhou H . The correlation between the use of the proton pump inhibitor and the clinical efficacy of immune checkpoint inhibitors in non-small cell lung cancer. J Oncol. (2022) 2022:1001796. doi: 10.1155/2022/1001796. PMID: 35855807 PMC9288308

[39] Arbour KC Mezquita L Long N Rizvi H Auclin E Ni A . Impact of baseline steroids on efficacy of programmed cell death-1 and programmed death-ligand 1 blockade in patients with non-small-cell lung cancer. J Clin Oncol. (2018) 36:2872–8. doi: 10.1200/JCO.2018.79.0006. PMID: 30125216

[40] Scott SC Pennell NA . Early use of systemic corticosteroids in patients with advanced NSCLC treated with nivolumab. J Thorac Oncol. (2018) 13:1771–5. doi: 10.1016/j.jtho.2018.06.004. PMID: 29935305

[41] Roboubi A Wasielewski E Bordier S Turlotte A Pavaut G Scherpereel A . Impact of corticosteroids on the efficacy of first-line pembrolizumab plus chemotherapy in patients with advanced non-small-cell lung cancer. Ther Adv Med Oncol. (2025) 17:17588359251318160. doi: 10.1177/17588359251318160. PMID: 39935765 PMC11811968

[42] Hendriks LEL Henon C Auclin E Mezquita L Ferrara R Audigier-Valette C . Outcome of patients with non-small cell lung cancer and brain metastases treated with checkpoint inhibitors. J Thorac Oncol. (2019) 14:1244–54. doi: 10.1016/j.jtho.2019.02.009. PMID: 30780002

[43] Hakozaki T Okuma Y Omori M Hosomi Y . Impact of prior antibiotic use on the efficacy of nivolumab for non-small cell lung cancer. Oncol Lett. (2019) 17:2946–52. doi: 10.3892/ol.2019.9899. PMID: 30854072 PMC6365976

[44] Derosa L Hellmann MD Spaziano M Halpenny D Fidelle M Rizvi H . Negative association of antibiotics on clinical activity of immune checkpoint inhibitors in patients with advanced renal cell and non-small-cell lung cancer. Ann Oncol. (2018) 29:1437–44. doi: 10.1093/annonc/mdy103. PMID: 29617710 PMC6354674

[45] Cortellini A Ricciuti B Facchinetti F Alessi JVM Venkatraman D Dall'Olio FG . Antibiotic-exposed patients with non-small-cell lung cancer preserve efficacy outcomes following first-line chemo-immunotherapy. Ann Oncol. (2021) 32:1391–9. doi: 10.1016/j.annonc.2021.08.1744. PMID: 34400292

[46] Kawachi H Yamada T Tamiya M Negi Y Goto Y Nakao A . Concomitant proton pump inhibitor use with pembrolizumab monotherapy vs immune checkpoint inhibitor plus chemotherapy in patients with Non-Small Cell Lung Cancer. JAMA Netw Open. (2023) 6:e2322915. doi: 10.1001/jamanetworkopen.2023.22915. PMID: 37432682 PMC10336622

[47] Guo Y Jia J Hao Z Yang J . Tislelizumab plus chemotherapy versus pembrolizumab plus chemotherapy for the first-line treatment of advanced non-small cell lung cancer: systematic review and indirect comparison of randomized trials. Front Pharmacol. (2023) 14:1172969. doi: 10.3389/fphar.2023.1172969. PMID: 37408759 PMC10318343

