# Supplementary material for: Real-world effectiveness of first-line immunotherapy with or without chemotherapy versus chemotherapy alone in advanced non-small cell lung cancer
Source: Front Immunol. 2026 May 12;17:1753591. doi: 10.3389/fimmu.2026.1753591 (PMC13201463; doi:10.3389/fimmu.2026.1753591)
Supplement: Supplementary file 2 [file Table1.docx]

Table S1. Baseline characteristics of patients in the propensity score-matched cohort.

| Characteristics | [ALL]  N=236 | chemotherapy N=118 | ICIs ± chemotherapy N=118 | *p* overall |
| --- | --- | --- | --- | --- |
| Sex, n (%) |  |  |  | 0.865 |
| Female | 42 (17.8%) | 20 (16.9%) | 22 (18.6%) |  |
| Male | 194 (82.2%) | 98 (83.1%) | 96 (81.4%) |  |
| Age(years), n (%) |  |  |  | 0.676 |
| <60 | 76 (32.2%) | 40 (33.9%) | 36 (30.5%) |  |
| ≥60 | 160 (67.8%) | 78 (66.1%) | 82 (69.5%) |  |
| BMI, n (%) |  |  |  | 0.437 |
| <18.5 | 20 (8.47%) | 10 (8.47%) | 10 (8.47%) |  |
| 18.5-24.9 | 141 (59.7%) | 66 (55.9%) | 75 (63.6%) |  |
| >25 | 75 (31.8%) | 42 (35.6%) | 33 (28.0%) |  |
| Nutrition Score, n (%) |  |  |  | 0.692 |
| <3 | 207 (87.7%) | 105 (89.0%) | 102 (86.4%) |  |
| ≥3 | 29 (12.3%) | 13 (11.0%) | 16 (13.6%) |  |
| Smoking, n (%) |  |  |  | 0.766 |
| No | 61 (25.8%) | 29 (24.6%) | 32 (27.1%) |  |
| Yes | 175 (74.2%) | 89 (75.4%) | 86 (72.9%) |  |
| Drinking, n (%) |  |  |  | 0.694 |
| No | 132 (55.9%) | 64 (54.2%) | 68 (57.6%) |  |
| Yes | 104 (44.1%) | 54 (45.8%) | 50 (42.4%) |  |
| Diabetes comorbidity,  n (%) |  |  |  | 1.000 |
| No | 208 (88.1%) | 104 (88.1%) | 104 (88.1%) |  |
| Yes | 28 (11.9%) | 14 (11.9%) | 14 (11.9%) |  |
| Cardiovascular diseases comorbidity, n (%) |  |  |  | 0.556 |
| No | 173 (73.3%) | 84 (71.2%) | 89 (75.4%) |  |
| Yes | 63 (26.7%) | 34 (28.8%) | 29 (24.6%) |  |
| Lung Disease Comorbidity, n (%) |  |  |  | 0.878 |
| No | 180 (76.3%) | 89 (75.4%) | 91 (77.1%) |  |
| Yes | 56 (23.7%) | 29 (24.6%) | 27 (22.9%) |  |
| T stage, n (%) |  |  |  | 0.515 |
| 1-2 | 122 (51.7%) | 64 (54.2%) | 58 (49.2%) |  |
| 3-4 | 114 (48.3%) | 54 (45.8%) | 60 (50.8%) |  |
| N stage, n (%) |  |  |  | 0.464 |
| 0-1 | 35 (14.8%) | 20 (16.9%) | 15 (12.7%) |  |
| 2-3 | 201 (85.2%) | 98 (83.1%) | 103 (87.3%) |  |
| Histological type, n (%) |  |  |  | 0.863 |
| Non-squamous | 151 (64.0%) | 77 (65.3%) | 74 (62.7%) |  |
| Squamous | 80 (33.9%) | 39 (33.1%) | 41 (34.7%) |  |
| Unknown | 5 (2.12%) | 2 (1.69%) | 3 (2.54%) |  |
| Liver metastasis, n (%) |  |  |  | 0.853 |
| No | 202 (85.6%) | 100 (84.7%) | 102 (86.4%) |  |
| Yes | 34 (14.4%) | 18 (15.3%) | 16 (13.6%) |  |
| Bone metastasis, n (%) |  |  |  | 1.000 |
| No | 127 (53.8%) | 64 (54.2%) | 63 (53.4%) |  |
| Yes | 109 (46.2%) | 54 (45.8%) | 55 (46.6%) |  |
| Brain metastasis, n (%) |  |  |  | 1.000 |
| No | 180 (76.3%) | 90 (76.3%) | 90 (76.3%) |  |
| Yes | 56 (23.7%) | 28 (23.7%) | 28 (23.7%) |  |
| PD-L1 expression,  n (%) |  |  |  | 0.891 |
| <1% | 40 (16.9%) | 20 (16.9%) | 20 (16.9%) |  |
| 1% ≤PD-L1 < 50% | 34 (14.4%) | 17 (14.4%) | 17 (14.4%) |  |
| PD-L1≥50% | 32 (13.6%) | 14 (11.9%) | 18 (15.3%) |  |
| Unknown | 130 (55.1%) | 67 (56.8%) | 63 (53.4%) |  |
| Dead, n (%) |  |  |  | 0.003 |
| No | 30 (12.7%) | 7 (5.93%) | 23 (19.5%) |  |
| Yes | 206 (87.3%) | 111 (94.1%) | 95 (80.5%) |  |
